# The Effects of Different Culture Modes on the Nutritional Quality of *Procambarus clarkii* and Mechanistic Insights: A Metabolomic Perspective

**DOI:** 10.3390/biology15110875

**Published:** 2026-06-02

**Authors:** Ting Liu, Juan Tian, Lang Zhang, Jianwu Chen, Yali Yu, Chen Tian, Jinhua Gan

**Affiliations:** 1 Yangtze River Fisheries Research Institute, Chinese Academy of Fishery Sciences, Wuhan 430223, China; cjliuting@yfi.ac.cn (T.L.); tianjuan@yfi.ac.cn (J.T.); zhanglang@yfi.ac.cn (L.Z.); chjw@yfi.ac.cn (J.C.); ylyu8811@yfi.ac.cn (Y.Y.); tianchen@yfi.ac.cn (C.T.); 2 Freshwater Fisheries Research Center, Chinese Academy of Fishery Sciences, Wuxi 214081, China

**Keywords:** *Procambarus clarkii*, culture mode, metabolomics, nutritional quality

## Abstract

*Procambarus clarkii*, a prominent aquaculture species, are mainly cultured through conventional modes: pond culture and rice–crayfish co-culture. In the present study, we proposed a novel industrial recirculating aquaculture system (RAS) for the culture of *Procambarus clarkii*. The nutritional quality of *Procambarus clarkii* under different culture modes was evaluated. The results indicated that industrial culture achieved optimal amino acid profiles and a higher level of flavor amino acids. Crayfish cultured in RAS also showed more balanced textures with moderate hardness and good springiness. Moreover, distinct crayfish metabolites were identified across different culture modes. The main differential metabolites include amino acids, peptides (and their analogs), organic acids and acyl carnitines. Industrial culture prioritized metabolites linked to flavor and rapid growth, while other culture modes enriched metabolites associated with ecological resilience and nutritional diversity. Overall, industrial culture displays great potential in improving the nutritional quality and regulating metabolic characteristics of red swamp crayfish.

## Simple Summary

Red swamp crayfish (*Procambarus clarkii*) is a major aquaculture species in China. We compared five culture modes, including a new industrial recirculating aquaculture system (RAS). Industrial culture yielded crayfish with superior amino acid profiles, higher flavor amino acids, and balanced muscle texture. Co-culture modes had richer polyunsaturated fatty acids like EPA and DHA. Metabolomics showed industrial culture favored flavor and fast-growth metabolites, while traditional modes enriched those for ecological resilience. Industrial RAS improves crayfish quality and enables year-round sustainable production, offering a key direction for the industry.

## 1. Introduction

Red swamp crayfish (*Procambarus clarkii*) originated in North America has become a key freshwater species with high economic value in China [1]. In 2024, China’s crayfish aquaculture production reached 3.45 million tons, representing over 98% of the global total. China exported 11,727 tons of crayfish products, with an export value of USD 116.05 million. The total output value of the crayfish industrial chain exceeded CNY 458 billion (approximately USD 64 billion) in 2023 [2]. Traditional crayfish culture modes include pond culture (PC), rice–crayfish co-culture (RC), crab–crayfish co-culture (CC) and lotus–crayfish co-culture (LC). Among them, rice–crayfish co-culture accounts for the largest proportion of the global crayfish production and has experienced remarkable growth in China over the past decade, particularly in the Jianghan Plain region [3]. The area dedicated to rice–crayfish co-culture has increased by 37.4% from 18.92 million hectares in 2020 to 26.00 million hectares in 2024 [2]. This expansion is attributed to the comprehensive benefits provided by the co-culture system, which stabilizes food synergy and favors crayfish over rice. While co-culture provides significant socioeconomic advantages, they face environmental issues such as water pollution, water resource wastage and methane emissions [4], as well as the pressing problem that the year-round supply of crayfish has been limited due to low survival and growth rate of crayfish in winter. Several studies have reported that low-temperature stimulation can cause severe damage to crayfish [5,6]. Crayfish metabolism will slow down significantly at low temperatures [7], reducing their ability to feed, digest food, and grow. Cold stress can delay or inhibit reproduction, reducing egg production and hatching success [8,9].

Driven by increasing demand for aquatic products, declining wild fish stocks and advancements in aquaculture technology, industrial aquaculture is rapidly growing in the global food industry, aiming to maximize production efficiency, reduce costs, and meet the growing need for sustainable protein sources. Industrial aquaculture systems have been applied for a variety of species, including sea cucumber (*Apostichopus japonicus*) [10], shrimp *Litopenaeus vannamei* [11], salmon, tilapia, and channel catfish (*Ictalurus punctatus*) [12]. Industrial aquaculture provides a reliable and consistent source of protein to meet the growing global food demand while reducing the impact on aquatic ecosystems and ensures a steady supply of aquatic products throughout the year [13]. Until now, however, there has been a lack of studies focusing on *P. clarkii* under industrial farming conditions. Extensive research has been conducted on traditional pond and rice–crayfish co-culture systems [14,15,16]. The potential for scaling up *P. clarkii* production using advanced technologies remains unexplored. For instance, little is known about the optimal stocking densities, water quality parameters, or feeding regimes required to maximize growth and survival rates under industrial farming. Additionally, the economic feasibility and environmental impacts of industrial *P. clarkii* farming have yet to be thoroughly investigated. Addressing these gaps could pave the way for more sustainable and efficient production methods, meeting the growing global demand for crayfish. Moreover, in recent years, with consumers’ growing awareness of health, more attention has been paid to the nutritional value and flesh quality of aquatic organisms. It was reported that farmed fish and shellfish are abundant in vital nutrients such as omega-3 fatty acids, vitamins, and minerals, contributing to the improvement of public health [17,18]. Several studies have investigated various culture modes concerning *P. clarkii*. A comparison was made between the nutritional value, non-specific immunity, and intestinal microflora of red swamp crayfish raised under different culture modes [19]. Rice field crayfish exhibited higher muscle nutritional value compared to those in the other groups. Compared with pond-reared *P. clarkii*, those from rice fields showed significantly better intestinal bacteria and muscle flavor [20]. However, the majority of studies have been focused on the rice–crayfish co-culture mode, without further evaluating comprehensive impacts of various culture modes on *P. clarkii*.

Recently, metabolomics has been widely applied, as it can sensitively reflect changes in physiological metabolism, nutrient synthesis, and flesh quality at the small molecule level. In aquatic products, metabolomic approaches have been successfully used to identify differential metabolites related to growth performance, nutritional composition, and stress adaptation under different rearing conditions [21,22,23]. These studies demonstrated that culture environment and feeding strategy significantly reshape metabolic profiles. However, no metabolomic studies have been reported to reveal the intrinsic mechanisms underlying nutritional quality differences in *Procambarus clarkii* under diverse culture modes.

Herein, we aim to provide a comprehensive evaluation on nutritional quality of crayfish under different culture modes. Except for the traditional crayfish culture modes, a novel industrial crayfish culture (IC) system was also employed. Muscle quality and nutritional profile of crayfish under five culture modes were assessed. Our study could help to optimize aquaculture techniques and enhance the market competitiveness of crayfish, meeting the demand of consumers for high-quality aquatic products.

## 2. Experimental Section

### 2.1. Experimental Design

Five crayfish culture modes were selected in this study: four common culture modes of *P. clarkii* in Hubei (PC, RC, CC, and LC) and a novel culture mode—IC. There was no difference in germplasm resources among all culture groups. All crayfish in the five culture modes were stocked at the same initial time and sampled simultaneously after identical rearing duration. PC was located at Qianjiang (110°47′52″ E, 30°23′47″ N), with a pond area of 2000 m^2^ (dimensions 50 m × 40 m, water depth 1.2–1.5 m) and 15 *P. clarkii* seedlings per m^2^. RC was located at Qianjiang (112°47′50″ E, 30°16′38″ N), with a pond area of 4000 m^2^ (dimensions 80 m × 50 m, water depth 0.8–1.0 m) and 20 *P. clarkii* seedlings per m^2^. CC was located at Wuhan (113°53′28″ E, 30°19′18″ N) with a pond area of 2000 m^2^ (dimensions 50 m × 40 m, water depth 1.2–1.5 m) and 13 *P. clarkii* and 3 Chinese mitten crabs, *Eriocheir sinensis*, seedlings per m^2^. LC was located at Honghu (113.44212° E, 30.08177° N) with a pond area of 1800 m^2^ (dimensions 45 m × 40 m, water depth 0.9–1.2 m) and 18 *P. clarkii* seedlings per m^2^. IC was conducted in an indoor flow-through culture system at the Hubei Crayfish Industry Research Institute, Qianjiang (Figure S1). The system contained shelter tubes and climbing nets to prevent inter-individual aggression and facilitate molting. Shelter tubes were made of PVC plastic, with a length of 20 cm, and an inner diameter of 5 cm, and were evenly distributed at 10 cm intervals along the bottom of each tank. Climbing nets were made of polyethylene (PE) material, with a mesh size of 1.0 cm × 1.0 cm, vertically fixed in the middle of each culture tank with a height of 30 cm. The crayfish were cultivated in culture tanks (depth 0.35 m, water volume 350 L) at a stocking density of 60 individuals/m^3^. During the cultivation period, the water flow rate was maintained at 30 L/h. Feces were removed using the siphon method. The water temperature ranged from 23 to 28 °C, with ammonia nitrogen levels below 0.05 mg/L, dissolved oxygen levels above 5 mg/L, and a pH range of 8.1 to 8.3. For the four traditional culture modes (PC, RC, CC, LC), water quality was not continuously measured or artificially regulated during the experiment. Crayfish in the industrial recirculating aquaculture system (IC) were fed with a formulated feed twice daily (at 8:00 and 18:00), at a daily feeding rate of 3–5% of crayfish body weight. The feed of *P. clarkii* during the culture period, with the main nutritional guarantee values shown in Table S1. For the four traditional culture modes (PC, RC, CC, LC), crayfish mainly fed on natural baits in the culture environment, and were supplemented with a small amount of compound feed once a day at a daily rate of 1–2% of body weight, following local conventional crayfish culture management.

### 2.2. Sample Collection

One hundred and fifty *P. clarkii* from five culture modes (*n* = 30 per group) were randomly collected. During collection, individuals with consistent body size and stable inter-molt stage were selected, and male and female samples were matched equally to reduce the influence of individual differences on experimental results. Prior to sampling, all crayfish were subjected to cold anesthesia by immersion in pre-cooled ice water until complete sedation. Then, the carapace of the crayfish was removed with sterilized scissors to extract the underlying muscle tissue. The extracted muscle tissue was then immediately placed in a pre-cooled container or on ice to preserve its biochemical integrity, and then all samples were rapidly frozen with liquid nitrogen, after which they were stored at −80 °C for subsequent analysis. Each of the five culture modes was established with three replicates to ensure the reliability of experimental data. All index determinations were conducted separately in a parallel group.

### 2.3. Muscle Properties and Nutritional Composition of Crayfish

The muscle texture, including hardness, chewiness, cohesiveness, adhesiveness and springiness, was measured by TVT-300XP Texture Analyzer (Perten Instruments, Stockholm, Sweden) equipped with a P/36R cylindrical probe. The pre-test speed, test speed and post-test speed were all set at 1 mm/s, the trigger force was 5 g, and the compression depth was 50% of the original sample height. Each sample was detected in six replicates. Further, 10 g of mixed crayfish muscle from each culture mode was measured for fatty acid and amino acid determination based on GB 5009.124-2016 [24] and GB 5009.168-2016 [25], respectively. Fatty acid and amino acid were determined using a Thermo Scientific TSQ9000 triple quadrupole mass spectrometer equipped with a Trace1300 gas chromatograph (Thermo Fisher Scientific, Waltham, MA, USA) and the amino acid automatic analyzer (L8900, Hitachi, Tokyo, Japan), respectively.

### 2.4. Analysis of Differential Metabolites of Crayfish Muscle Under Five Culture Modes

Metabolite extraction was performed by treating muscle tissues with 1 mL of pre-chilled methanol/acetonitrile/water solution (2:2:1, *v*/*v*) using ice bath-assisted sonication for 1 h. Following this treatment, the mixture underwent incubation at −20 °C for 1 h and subsequent centrifugation (16,000× *g*, 4 °C, 20 min). The resulting supernatant was collected in sampling vials for subsequent LC-MS analysis.

The liquid chromatography system (Shimadzu Nexera X2 LC-30AD, Shimadzu Corporation, Kyoto, Japan) was coupled to a triple quadrupole mass spectrometer (5500 QTRAP, AB Sciex, Framingham, MA, USA) using an Acquity UPLC HSS T3 column (2.1 × 50 mm, 1.8 μm; Waters, Milford, MA, USA). Chromatographic separation was achieved at 40 °C with a 200 μL/min flow rate, employing a binary mobile phase: 0.1% formic acid aqueous solution (Solvent A) and 100% acetonitrile (Solvent B). The gradient program initiated with 100% Solvent A (0–2.5 min), followed by linear transitions to 70% Solvent A (2.5–11.5 min) and 0% Solvent A (11.5–12.5 min). After a 5.4 min isocratic phase, the system reverted to initial conditions over 0.1 min and equilibrated for 2.5 min. Samples (5 μL injection volume) were analyzed sequentially using both electrospray ionization (ESI) modes. The full scan mass range was *m*/*z* 50–1000. Collision energy was optimized within 10–45 eV for fragment ion fragmentation. Data-dependent acquisition (DDA) mode was used for mass spectrum data collection to obtain comprehensive metabolite information.

### 2.5. Metabolomics Data Analysis

Significant metabolites were identified through combined multivariate and univariate statistical approaches. Multivariate screening employed variable importance in projection (VIP) scores derived from orthogonal projections to latent structures–discriminant analysis (OPLS-DA), while univariate assessment utilized two-tailed Student’s *t*-tests (*p*-value < 0.05). For multi-group comparisons, *p*-values were determined through one-way ANOVA. All raw data were firstly assessed for normal distribution and homogeneity of variance. ANOVA was adopted for datasets conforming to normal distribution and satisfied homogeneity of variance, then Duncan’s multiple range test was applied for multiple comparisons at the significance level of *p* < 0.05. Meanwhile, PERMANOVA was conducted to statistically confirm significant metabolic discrepancies among different culture modes (Table S2). Compounds meeting dual thresholds (VIP > 1, *p* < 0.05) were classified as statistically significant and subsequently subjected to hierarchical clustering analysis using the R package (R version 4.3.1).

### 2.6. Statistical Analysis

Statistical analyses for significant differences were conducted using IBM SPSS Statistics 20. All sample data are expressed as mean ± standard deviation (SD), with *p*-values < 0.05 considered indicative of statistical significance. Data visualization and computational processing were carried out using OriginPro 2019 (OriginLab Corporation, Northampton, MA, USA) and Microsoft Excel 2016 software platforms.

## 3. Results and Discussion

### 3.1. Effects of Culture Mode on the Texture of P. clarkii Muscle

**Figure 1:**
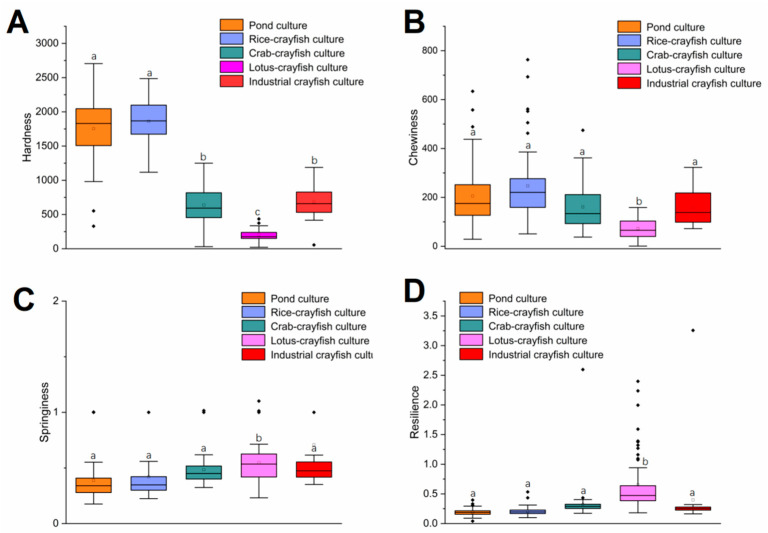
Effects of different culture modes on textural properties of red swamp crayfish. (**A**) Hardness. (**B**) Chewiness. (**C**) Springiness. (**D**) Resilience. Each subgraph marked with different superscript letters indicate significant differences (*p* < 0.05), whereas those sharing identical superscript letters show no statistically significant differences (*p* > 0.05).

The textural properties of *P. clarkii* muscle under different culture modes were presented in Figure 1A–D. Hardness and chewiness of *P. clarkii* muscle were higher under pond culture and rice–crayfish culture mode (Figure 1A,B). Springiness and resilience of *P. clarkii* muscle were higher under lotus–crayfish culture mode (Figure 1C,D). These differences are largely attributed to the combined influences of activity level disparities and divergent environmental regulation patterns across culture modes. Crayfish reared under pond culture (PC) and rice–crayfish co-culture (RC) habitats generally present higher spontaneous activity. Such behavioral distinctions are closely associated with the low-density open water conditions in PC and the complex field surroundings in RC. In line with previous studies, long-term differences in daily activity patterns can further alter muscle tissue physical properties, thereby contributing to the evident variations in muscle hardness and chewiness [26]. However, the unstable protein supply led to relatively low springiness and resilience [27]. In the lotus–crayfish co-culture (LC) mode, the shallow water lotus environment provided abundant organic detritus and habitat structures. This is associated with the improvement of water nutrient availability by lotus root exudates, which in turn regulates the muscle protein structure. Differences in burrowing and climbing behaviors may partially affect muscle fiber characteristics, which could further contribute to variations in springiness and resilience of crayfish muscle tissues [28]. The crab–crayfish co-culture (CC) and industrial recirculating aquaculture system (IC) modes showed a balanced texture characteristic. In the CC mode, the competitive pressure between crabs and crayfish led to a moderate exercise intensity of the crayfish. In the IC mode, water quality indicators including dissolved oxygen above 5 mg/L and ammonia nitrogen below 0.05 mg/L, together with steady water flow at 30 L/h, were precisely maintained. Such confined rearing conditions may limit spontaneous activity of crayfish. Meanwhile, sufficient and stable feed provision could help sustain muscle chewiness and springiness. These results suggest that environmental rearing conditions and nutritional supply jointly exert combined effects on crayfish muscle texture properties [29,30].

The IC mode realizes the precise regulation of muscle quality through artificial control. On the one hand, the stable aquatic environmental conditions including favorable water temperature, sufficient dissolved oxygen and low ammonia nitrogen levels may help alleviate metabolic stress in crayfish [31,32]. Such favorable rearing environments are likely to slow down muscle protein degradation, which could further contribute to maintaining the relatively intact structure of muscle fibers. On the other hand, the formulated feed with a balanced nutrition ratio provides sufficient amino acids and energy substances for muscle synthesis, especially the addition of ingredients that promote muscle elasticity, which helps to maintain the springiness and chewiness of the muscles. However, the limited movement space in the IC mode reduces the exercise intensity of the crayfish, leading to a slightly lower muscle fiber density and hardness compared to the PC and RC modes.

### 3.2. Effects of Culture Mode on Amino Acid Content of P. clarkii Muscle

Seventeen amino acid contents of *P. clarkii* muscle under five culture modes were analyzed, including seven essential amino acids necessary for human nutrition, two semi-essential amino acids, and eight non-essential amino acids for humans. The contents and compositions of amino acid were shown in Table 1. Industrial culture (IC) exhibited the highest TAA (15.3%), while pond culture (PC), rice–crayfish (RC), lotus–crayfish (LC), and crab–crayfish co-culture (CC) showed comparable TAA levels (13.8–14.1%). IC also dominated total EAA content (6.04%), followed by PC (5.48%), with minimal differences among RC, LC, and CC (5.39–5.45%). Isoleucine (Ile), leucine (Leu), phenylalanine (Phe), and lysine (Lys) were consistently highest in IC. Methionine (Met) showed minimal variation among all culture modes, though IC slightly exceeded others. Threonine (Thr), valine (Val), and histidine (His) exhibited no significant differences. IC also ranked highest NEAA (6.89%), while PC had the lowest (5.88%). Glutamic acid (Glu) and aspartic acid (Asp), which were key contributors to umami taste, were highest in IC, suggesting enhanced flavor potential in industrial culture crayfish. To comprehensive analysis of flavor attributes, flavor amino acid contents in the muscles of *P. clarkii* cultured under different modes were presented in Table 2. Industrial culture (IC) exhibited the highest umami amino acid content (3.44%). This aligns with elevated glutamic acid and aspartic acid levels in IC (Table 1). IC also dominated sweet amino acids (3.49%), driven by higher glycine and alanine (Table 1). IC also led in bitter amino acids (6.55%), consistent with its higher total essential amino acids (Table 1). Elevated bitter amino acids in IC may slightly offset its superior umami and sweetness but are balanced by higher total flavor complexity.

The total amino acid (TAA) and essential amino acid (EAA) contents in the crayfish muscles in the IC mode were significantly higher than those in other modes. Additionally, the total content of umami amino acids (glutamic acid: 3.44%, aspartic acid: 2.89%) and sweet amino acids (glycine: 1.82%, alanine: 1.67%) reached 6.93%, which was 18.7% higher than that in the RC mode (Table 2). This advantage of the IC mode stems from dual regulations: firstly, the addition of umami precursors such as yeast extract in the feed promotes the synthesis of glutamyl peptides; secondly, the strict control of environmental parameters reduces metabolic stress, and the protein decomposition induced by cortisol is decreased, allowing more amino acids to be used for muscle synthesis.

In contrast, the rice fields, lotus ponds, and crab ponds provide a variety of organisms for the crayfish to feed on. These natural baits contain different types and proportions of amino acids, which enrich the amino acid composition of the crayfish muscles [33,34]. However, the content and quality of natural baits are affected by environmental conditions, resulting in unstable amino acid intake of the crayfish. It is worth noting that the content of bitter amino acids in the IC mode was relatively high (6.55%), but the ratio of bitter amino acids to umami and sweet amino acids (1:1.06) was still within the flavor balance range, without negatively affecting the overall taste quality. This is consistent with the research results of Wang et al. (2023) [19] in the comparison of different crayfish culture modes, where the RC and CC modes showed comparable efficacy in amino acid accumulation.

### 3.3. Effects of Culture Mode on Fatty Acid Content of P. clarkii Muscle

A total of 37 fatty acids were determined in this study, with 22 types observed in the muscle of *P. clarkii* cultured under the five culture modes. These included 10 saturated fatty acids (SFAs), 5 monounsaturated fatty acids (MUFAs), and 7 polyunsaturated fatty acids (PUFAs). The contents and compositions of fatty acid were shown in Table 3. The results showed that ∑SFAs were highest in pond culture (PC: 52.26%) and lowest in industrial culture (IC: 38.44%). C16:0 (palmitic acid) was the most abundant SFA in all modes, with PC showing the highest proportion (32.51%) and IC the lowest (21.90%). C18:0 (stearic acid) followed a similar trend, peaking in PC (17.11%) and declining in RC (12.29%), LC (12.52%), CC (13.09%), and IC (14.10%). Trace amounts of odd-chain SFAs (C13:0, C15:0) were detected in LC and CC modes but absent in PC and IC. The dominance of saturated fatty acids (SFAs) in pond culture (PC) may be partly related to their lipid metabolic patterns and dietary sources within pond habitats. In such aquatic environments, organic detritus and ambient microbial activities could facilitate the relative accumulation of SFAs [35]. In contrast, the lower SFA content in industrial culture (IC) likely stems from formulated feeds enriched in unsaturated fatty acids, which are more easily incorporated into muscle tissue and less prone to deposition as storage lipids [36]. The near-absence of odd-chain SFAs (C13:0, C15:0) in PC and IC, compared to their trace presence in co-cultures (LC, CC), may imply that such rare fatty acids are probably derived from algae or microbial metabolites, which are more abundant in ecologically diversified culture environments. The complex nutrient cycling among aquatic plants, crustaceans and microorganisms in these systems may further enrich diverse lipid sources [37].

∑MUFAs were highest in industrial culture (IC: 25.24%) and lowest in lotus–crayfish co-culture (LC: 19.35%). C18:1 (oleic acid) dominated MUFA profiles under all modes, with IC showing the highest proportion (22.08%) and LC the lowest (12.96%). C16:1 (palmitoleic acid) was notably elevated in RC (2.81%) and LC (4.96%). ∑PUFAs varied significantly, with LC (37.76%) and CC (35.98%) displaying the highest levels, while PC (22.82%) was the lowest. C20:5 (EPA) and C22:6 (DHA) were highest in LC (14.85% and 5.01%, respectively), reflecting the ecological richness of lotus–crayfish habitats. IC and PC showed comparatively lower EPA (10.73% and 10.01%) and DHA (1.73% and 1.54%). C18:2 (linoleic acid) was highest in CC (14.37%), suggesting crab–crayfish interactions may enhance its retention. C20:4 (arachidonic acid) peaked in LC (7.61%) and PC (7.09%), indicating mode-specific lipid metabolism. Co-culture modes (LC, RC, CC) exhibited lower n-6/n-3 PUFA ratios due to higher omega-3 content, while PC and IC showed elevated ratios, reflecting plant-based feed dominance. Environmental complexity emerges as a critical driver of PUFA diversity [38]. Co-cultures, with their heterogeneous habitats (rice paddies, lotus beds, mixed crustacean communities), offer a various sources of lipid, promoting the accumulation of diverse PUFAs. IC, with its controlled diet and simplified environment, prioritizes MUFAs, which might be a trade-off between predictable lipid deposition and nutritional diversity.

The fatty acid profile of crayfish muscles is mainly determined by the lipid source in the feed, habitat and the lipid metabolism ability of the crayfish [39,40]. In the PC mode, the lipid source is mainly endogenous synthesis and detrital organic matter. The microorganisms in the pond decompose organic matter to produce a large amount of SFAs [41], which are then absorbed and accumulated by the crayfish, resulting in a high content of SFAs in the muscles. In the IC mode, the formulated feed is rich in MUFAs, which are easily absorbed and incorporated into the muscle tissue by the crayfish. At the same time, the controlled environment in the IC mode reduces the lipid oxidation of the crayfish [42], maintaining the stability of MUFAs. In the co-culture modes, the complex ecological environment provides a variety of lipid sources for the crayfish. For example, the algae and aquatic plants in the LC mode are rich in PUFAs (such as EPA and DHA), which are more easily absorbed by the crayfish and accumulated in the muscles, resulting in a high content of PUFAs.

### 3.4. Differential Metabolite Profile in Crayfish Muscle Under Different Culture Modes

**Figure 2:**
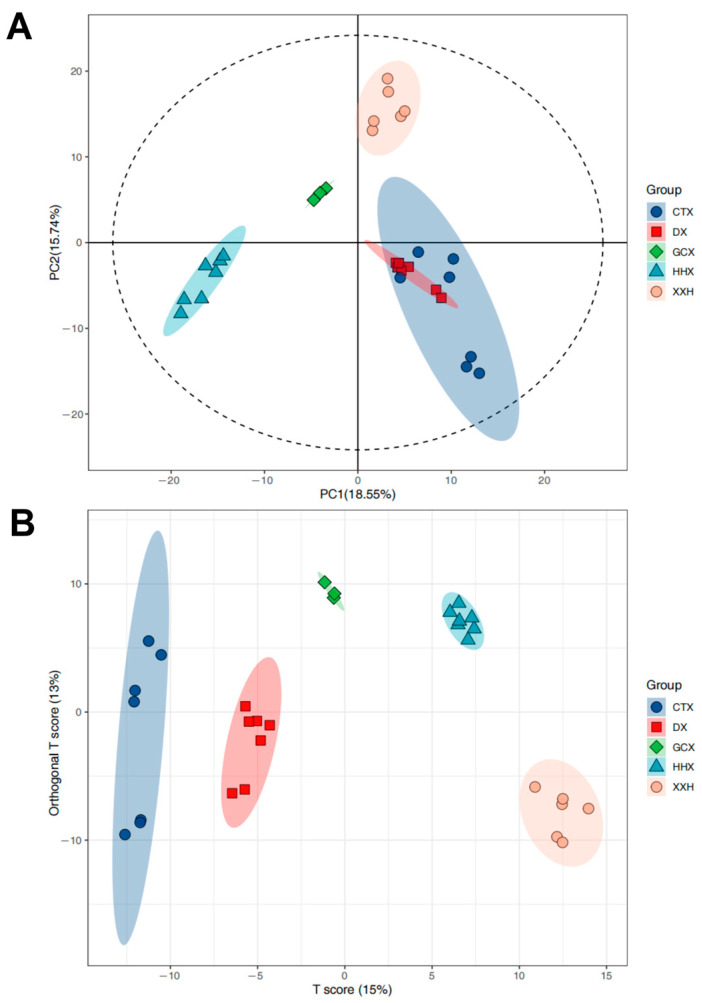
PCoA scores plot of differential metabolites from crayfish under different culture modes (**A**) and OPLS-DA model of differential metabolites analysis of crayfish samples; The model evaluated with a goodness of fit (R2Y, 0.993) and goodness of prediction (Q2Y, 0.93) (**B**). (CTX: pond culture; DX: rice field co-culture; GCX: industrial culture; HHX: lotus–crayfish co-culture; XXH: crab–crayfish co-culture).

**Figure 3:**
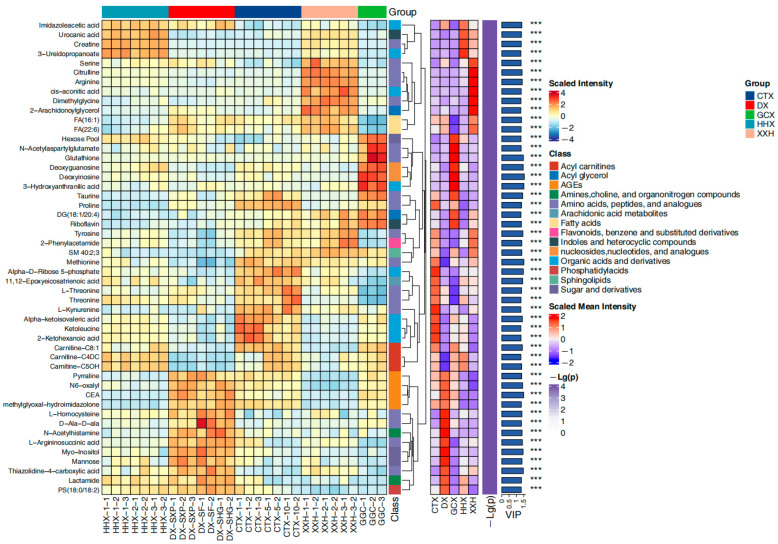
Cluster heat map of differential metabolites for crayfish samples under different culture modes. (CTX: pond culture; DX: rice field co-culture; GCX: industrial culture; HHX: lotus–crayfish co-culture; XXH: crab–crayfish co-culture). *** indicates VIP > 1.

**Figure 4:**
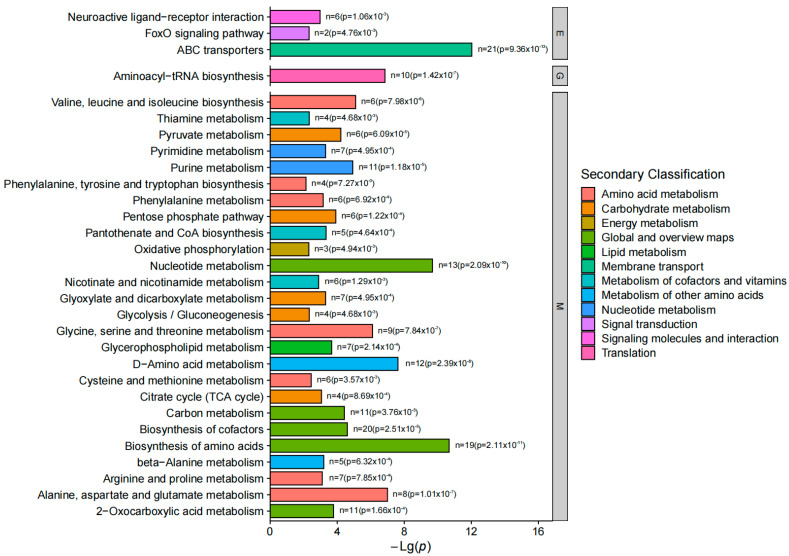
KEGG enrichment pathway bar chart shows the top 30 pathway for differential metabolites of crayfish samples.

A total of 558 metabolites using both electrospray ionization (ESI) modes were identified in crayfish muscle under different culture modes. The overview patterns of the metabolites difference were shown in the PCA score plots (R2X = 0.531, Figure 2A). The PCA score plots indicated that there were obvious difference in metabolites among crab–crayfish co-culture (CC), lotus–crayfish co-culture (LC) and industrial crayfish culture (IC) but few differences were found between pond monoculture (PC) and rice–crayfish co-culture (RC). OPLS-DA was used to further illustrate the differences in metabolites among crayfish muscle under different culture modes. The R2Y was 0.993 and Q2Y was 0.93 (Figure 2B), indicating strong predictability and reliability of the model. Heat maps were generated to systematically visualize and interpret the variations in metabolite profiles across the five culture modes (Figure 3). Intense red signals representing abundant amino acids were prominently observed in IC group samples, which was highly consistent with its higher total contents of sweet amino acids recorded in Table 1. Organic acids and their derivatives, which are closely associated with ambient organic debris circulation and plant–microbe interaction processes [43], were found to be markedly accumulated in LC, PC and CC groups. By contrast, the RC group was characterized by the predominant enrichment of amines and choline as well as other organonitrogen metabolites. From the heatmap, the industrial culture tended to accumulate more metabolites closely related to muscle formation and growth metabolism. In comparison, metabolites enriched in co-culture modes were more inclined to correlate with habitat adaptability and diverse nutritional composition characteristics. To clarify the biochemical mechanisms behind the observed variations in metabolite profiles under five culture modes, metabolic pathway analysis was conducted. The metabolites were assigned to KEGG pathways based on their roles in biochemical reactions (Figure 4). They mainly involved four types of biological metabolic pathways, biosynthesis of amino acids, ABC transporters, D-amino acid metabolism and aminoacyl-tRNA biosynthesis. The metabolite class distribution across these four KEGG pathways highlights distinct biochemical strategies in different culture modes. For crayfish with different environmental adaptability requirements, the simultaneous activation of multiple metabolic pathways confers prominent physiological advantages. For instance, co-culture groups significantly activated glycolysis pathways, which are essential for energy metabolism, nucleotide synthesis and coenzyme formation [44]. Various exogenous carbohydrate substances and natural nutrient substrates derived from complex aquatic environments provide abundant metabolic precursors, effectively diversifying carbohydrate metabolic processes. Such metabolic alteration helps optimize energy supply efficiency and nutrient utilization efficiency, and supplies richer metabolic intermediates to support normal growth and development. In addition, different culture conditions could notably facilitate amino acid biosynthesis, including arginine and proline metabolism. These pathways are vital for in vivo protein synthesis, somatic growth and tissue repair of crayfish [45]. Meanwhile, secondary metabolic pathways represented by cysteine and methionine metabolism were obviously enriched in LC groups. These pathways participate in the synthesis of antioxidant substances such as glutathione, which can alleviate oxidative damage, relieve environmental stress responses and further improve the overall environmental adaptability of crayfish [46]. In contrast, the IC group mainly activated growth and flavor-related metabolic pathways to prioritize growth performance and edible quality.

### 3.5. Comparison and Analysis of Differential Metabolites of Crayfish Muscle Under Industrial Culture Modes

**Figure 5:**
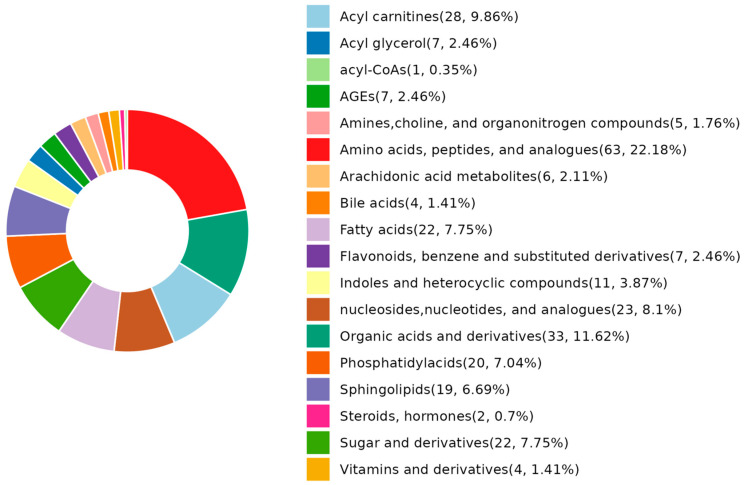
Differential metabolites classification ring diagram.

The distinct metabolic profile of *P. clarkii* in industrial-scale culture (IC) unveils a sophisticated interplay between artificial rearing conditions, dietary inputs, and adaptive physiological responses. Among the metabolites detected, amino acids, peptides and analogs (63), organic acids and derivatives (33) and acyl carnitines (28) had the most species of differential metabolites (Figure 5). The 17 significantly up-regulated metabolites, including glutathione (GSH), S-D-lactoylglutathione, taurine, riboflavin (B2), thiamine (B1), L-5-methyltetrahydrofolate, deoxyguanosine, 7-methylguanosine, N-L-γ-glutamyl-L-isoleucine, 5-hydroxy-L-tryptophan, DG (18:1/20:4) and PE (P18:0/22:6), in IC collectively reinforce its advantages in nutritional quality and productivity. Antioxidants such as glutathione (GSH), S-D-lactoylglutathione, and taurine are elevated to counteract oxidative stress inherent in sterile RAS. Unlike co-culture modes, IC’s pathogen-limited environment may weaken such natural defensive metabolic characteristics. GSH may alleviate excess ROS accumulation under relatively active metabolic conditions, while S-D-lactoylglutathione participates in the detoxification of methylglyoxal derived from moderate protein catabolism. Additionally, taurine could contribute to the maintenance of cell membrane integrity [47,48]. This adaptive response ensures energy is directed to muscle growth rather than repairing oxidative damage. Cofactors (riboflavin B2, thiamine B1, L-5-methyltetrahydrofolate) and nucleic acid derivatives (deoxyguanosine, 7-methylguanosine) further reflect IC’s metabolic shift toward efficiency. Riboflavin and thiamine fuel ATP production and nutrient catabolism, supporting the high protein demands of IC feeds, while L-5-methyltetrahydrofolate accelerates amino acid metabolism and nucleic acid synthesis, which is critical for muscle deposition [49]. Flavor-related metabolites, including N-L-γ-glutamyl-L-isoleucine and 5-hydroxy-L-tryptophan, directly enhance umami perception and taste complexity, aligning with IC’s highest umami amino acid content. Membrane lipids DG (18:1/20:4) and PE (P18:0/22:6) also optimize texture and juiciness, compensating for lower total DHA [36].

Conversely, 9 down-regulated metabolites reflect the metabolic limitations of the IC. Betaine, L-homoserine, and L-argininosuccinate, that are microbially derived metabolites abundant in RC/LC, are reduced in IC. This trend is presumably attributed to the relatively sterile rearing conditions in industrial systems, which may weaken microbial symbiosis and thereby reduce microbial-derived osmolyte and amino acid synthesis [18]. Although artificial feed supplementation could compensate for certain amino acid deficiencies, the reduction in microbe-associated metabolites may moderately lower overall metabolic diversity in IC crayfish. Lower DHA and palmitoleic acid levels may indicate nutritional constraints of formulated feeds; such algal-derived PUFAs were naturally scarce in plant-based feed ingredients. Collectively, the distinct metabolic signature observed in IC crayfish represents a specialized physiological adaptation to intensive rearing conditions.

## 4. Conclusions

The nutritional quality of red swamp crayfish under five culture modes was compared in our current study. The crayfish cultured in RAS achieved more balanced textures and higher level of flavor amino acids, while those under co-culture modes showed superior fatty acid content. The metabolomic analysis suggested metabolites linked to flavor and rapid growth were enriched in industrial culture, while other culture modes enriched metabolites associated with ecological resilience and nutritional diversity. This study provided a comprehensive understanding of the impact of various culture modes on crayfish quality. The findings can directly guide the crayfish aquaculture industry and help farmers optimize their culture practices to produce crayfish with desired nutritional characteristics, meeting the diverse needs of consumers. Continued innovation in feed formulation and industrial system design will be critical to fully realize the potential of industrial crayfish culture.

## Figures and Tables

**Table 1:** Amino acid compositions in the muscles of *P. clarkii* cultured under different modes (% fresh sample, *n* = 3).

	PC	RC	LC	CC	IC
Asp	1.28 ± 0.21 ^a^	1.27 ± 0.09 ^a^	1.27 ± 0.06 ^a^	1.26 ± 0.11 ^a^	1.37 ± 0.06 ^b^
Thr *	0.62 ± 0.10	0.62 ± 0.05	0.63 ± 0.03	0.62 ± 0.06	0.67 ± 0.03
Ser	0.59 ± 0.10	0.60 ± 0.04	0.61 ± 0.03	0.61 ± 0.05	0.65 ± 0.03
Glu	1.88 ± 0.33 ^a^	1.90 ± 0.17 ^a^	1.89 ± 0.10 ^a^	1.88 ± 0.19 ^a^	2.07 ± 0.10 ^b^
Gly	0.64 ± 0.08	0.70 ± 0.03	0.66 ± 0.04	0.64 ± 0.05	0.69 ± 0.03
Ala	1.00 ± 0.17 ^a^	1.13 ± 0.06 ^b^	1.07 ± 0.08 ^a^	1.01 ± 0.10 ^a^	1.13 ± 0.05 ^b^
Cys	0.12 ± 0.02	0.13 ± 0.01	0.12 ± 0.02	0.11 ± 0.02	0.14 ± 0.01
Val *	0.67 ± 0.10	0.66 ± 0.04	0.66 ± 0.02	0.66 ± 0.06	0.70 ± 0.03
Met *	0.20 ± 0.03	0.20 ± 0.04	0.20 ± 0.02	0.19 ± 0.04	0.23 ± 0.02
Ile *	0.74 ± 0.24 ^a^	0.71 ± 0.13 ^a^	0.72 ± 0.08 ^a^	0.72 ± 0.16 ^a^	0.86 ± 0.10 ^b^
Leu *	1.57 ± 0.33 ^a^	1.55 ± 0.16 ^a^	1.56 ± 0.10 ^a^	1.54 ± 0.19 ^a^	1.73 ± 0.13 ^b^
Tyr	0.46 ± 0.10	0.44 ± 0.03	0.44 ± 0.04	0.44 ± 0.07	0.48 ± 0.03
Phe *	0.59 ± 0.17	0.59 ± 0.10	0.60 ± 0.06	0.59 ± 0.11	0.68 ± 0.05
Lys *	1.08 ± 0.16 ^a^	1.08 ± 0.08 ^a^	1.09 ± 0.05 ^a^	1.07 ± 0.09 ^a^	1.16 ± 0.06 ^b^
His ^#^	0.30 ± 0.12	0.26 ± 0.04	0.23 ± 0.02	0.25 ± 0.05	0.29 ± 0.03
Arg ^#^	1.87 ± 0.33 ^a^	1.87 ± 0.15 ^a^	1.93 ± 0.10 ^a^	1.91 ± 0.18 ^a^	2.05 ± 0.09 ^b^
Pro	0.37 ± 0.05	0.33 ± 0.02	0.33 ± 0.02	0.33 ± 0.03	0.35 ± 0.01
∑EAA	5.48 ± 1.14 ^a^	5.41 ± 0.54 ^a^	5.45 ± 0.36 ^a^	5.39 ± 0.70 ^a^	6.04 ± 0.40 ^b^
∑NEAA	5.88 ± 0.94 ^a^	6.50 ± 0.42 ^b^	6.38 ± 0.36 ^b^	6.27 ± 0.61 ^b^	6.89 ± 0.31 ^c^
TAA	13.9 ± 2.77 ^a^	14.1 ± 1.15 ^a^	13.9 ± 1.01 ^a^	13.8 ± 1.54 ^a^	15.3 ± 0.83 ^b^
EAA/ NEAA	0.93 ± 0.04	0.83 ± 0.03	0.85 ± 0.01	0.86 ± 0.03	0.88 ± 0.02
EAA/ TAA	0.39 ± 0.00	0.38 ± 0.01	0.39 ± 0.00	0.39 ± 0.01	0.40 ± 0.01

Data were expressed as means ± standard deviations. Values in the same row with no common superscript letters are significantly different (*p* < 0.05), while values with the same letter or without a superscript letter in the same row are not significantly different (*p* > 0.05). PC: pond culture. RC: rice–crayfish co-culture. LC: lotus–crayfish co-culture. CC: crab and crayfish co-culture. IC: industrial crayfish culture. * essential amino acid for humans; ^#^ semi-essential amino acid. ΣEAA is the total amount of essential amino acids, ΣNEAA is the total amount of non-essential amino acids, and TAA is the total amino acid. Trp is destroyed during acidolysis without detection.

**Table 2:** Flavor-related amino acid contents in the muscles of *P. clarkii* cultured under different modes (% fresh sample, *n* = 3).

	PC	RC	LC	CC	IC
∑SOAA	3.16 ± 0.54 ^a^	3.16 ± 0.26 ^a^	3.16 ± 0.15 ^a^	3.14 ± 0.30 ^a^	3.44 ± 0.17 ^b^
∑SWAA	3.21 ± 0.49 ^a^	3.39 ± 0.19 ^b^	3.28 ± 0.18 ^a^	3.19 ± 0.29 ^a^	3.49 ± 0.14 ^c^
∑BIAA	5.94 ± 1.33 ^a^	5.84 ± 0.60 ^a^	5.90 ± 0.40 ^a^	5.87 ± 0.78 ^a^	6.55 ± 0.43 ^b^
∑FAA	6.68 ± 1.12 ^a^	6.88 ± 0.50 ^b^	6.82 ± 0.37 ^b^	6.69 ± 0.64 ^a^	7.31 ± 0.33 ^c^
∑TAA	13.9 ± 2.77 ^a^	14.1 ± 1.15 ^a^	13.9 ± 1.01 ^a^	13.8 ± 1.54 ^a^	15.3 ± 0.83 ^b^

Data were expressed as means ± standard deviations. Values in the same row with no common superscript letters are significantly different (*p* < 0.05), while values with the same letter or without a superscript letter in the same row are not significantly different (*p* > 0.05). PC: pond culture. RC: rice–crayfish co-culture. LC: lotus–crayfish co-culture. CC: crab and crayfish co-culture. IC: industrial crayfish culture. ΣSOAA represents the total amount of delicious amino acids (Asp, Glu); ΣSWAA represents the total amount of sweet amino acids (Thr, Gly, Ala, Ser, Pro), ΣBIAA represents the total amount of bitter amino acids (Leu, Met, Val, Ile, Phe, His, Arg), ΣFAA represents the total amount of taste-active amino acids (Arg, Asp, Glu, Gly, Ala), and ΣTAA is the total amino acid.

**Table 3:** Fatty acid compositions in the muscles of *P. clarkii* cultured under different modes (% fresh sample, *n* = 3).

Fatty Acid (%)	PC	RC	LC	CC	IC
C12:0	0.20 ± 0.03 ^a^	0.17 ± 0.01 ^a^	0.10 ± 0.01 ^b^	0.29 ± 0.00 ^a^	0.28 ± 0.06 ^a^
C13:0	nd	nd	0.02 ± 0.00	nd	nd
C14:0	0.88 ± 0.14 ^a^	0.72 ± 0.07 ^a^	0.69 ± 0.09 ^b^	0.71 ± 0.02 ^b^	0.73 ± 0.11 ^b^
C15:0	0.54 ± 0.08 ^a^	0.84 ± 0.18 ^b^	1.37 ± 0.22 ^c^	0.71 ± 0.10 ^b^	0.47 ± 0.02 ^a^
C16:0	32.51 ± 5.85 ^a^	25.68 ± 5.28 ^b^	23.98 ± 2.36 ^b^	26.06 ± 4.40 ^b^	21.90 ± 3.17 ^b^
C17:0	0.63 ± 0.06 ^a^	0.89 ± 0.15 ^a^	1.32 ± 0.22 ^b^	0.45 ± 0.33 ^a^	0.57 ± 0.33 ^a^
C18:0	17.11 ± 3.70 ^a^	12.29 ± 3.68 ^b^	12.52 ± 1.02 ^b^	13.09 ± 3.18 ^b^	14.10 ± 2.03 ^b^
C20:0	0.39 ± 0.02	0.43 ± 0.02	0.45 ± 0.06	0.31 ± 0.03	0.35 ± 0.02
C21:0	nd	nd	0.09 ± 0.00	0.03 ± 0.00	0.04 ± 0.00
C22:0	nd	nd	0.17 ±0.03 ^a^	0.07 ± 0.02 ^b^	nd
∑SFA	52.26 ± 3.62 ^a^	41.02 ± 5.27 ^b^	40.71 ± 6.33 ^b^	41.72 ± 4.26 ^b^	38.44 ± 3.59 ^b^
C15:1	0.14 ± 0.02	0.12 ± 0.00	0.08 ± 0.00	0.17 ± 0.02	0.13 ± 0.00
C16:1	1.54 ± 0.20 ^a^	2.81 ± 0.59 ^a^	4.96 ± 0.64 ^b^	2.35 ± 0.44 ^a^	1.51 ± 0.11 ^a^
C17:1	0.18 ± 0.03 ^a^	0.30 ± 0.07 ^a^	0.92 ± 0.16 ^b^	0.22 ± 0.06 ^a^	0.24 ± 0.02 ^a^
C18:1	17.68 ± 3.25 ^a^	19.30 ± 1.69 ^a^	12.96 ± 1.11 ^b^	16.52 ± 2.09 ^a^	22.08 ± 0.47 ^c^
C20:1	0.93 ± 0.21 ^a^	0.92 ± 0.22 ^a^	0.43 ± 0.07 ^b^	nd	1.28 ± 0.09 ^c^
∑MUFA	20.47 ± 2.97 ^a^	23.46 ± 3.77 ^a^	19.35 ± 6.51 ^a^	19.26 ± 3.22 ^a^	25.24 ± 4.83 ^b^
C18:2	7.92 ± 1.60 ^a^	9.60 ± 0.93 ^b^	8.88 ± 1.17 ^b^	14.37 ± 2.19 ^c^	9.71 ± 0.65 ^b^
C18:3	0.10 ± 0.02	0.20 ± 0.07	0.23 ± 0.02	0.15 ± 0.03	0.13 ± 0.00
C20:2	0.72 ± 0.21	0.70 ± 0.09	0.89 ± 0.17	0.72 ± 0.12	0.80 ± 0.06
C20:3	0.16 ± 0.04	0.26 ± 0.06	0.29 ± 0.03	0.14 ± 0.02	0.18 ± 0.02
C20:4	7.09 ± 4.09 ^a^	5.27 ± 5.02 ^b^	7.61 ± 0.85 ^a^	4.67 ± 0.79 ^b^	5.01 ± 0.42 ^b^
C20:5	10.01 ± 2.33 ^a^	11.64 ± 0.58 ^a^	14.85 ± 1.92 ^b^	12.79 ± 1.66 ^a^	10.73 ± 0.59 ^a^
C22:6	1.54 ± 0.64 ^a^	3.45 ± 0.32 ^b^	5.01 ± 1.26 ^b^	3.14 ± 0.42 ^b^	1.73 ± 0.19 ^a^
∑PUFA	22.82 ± 2.16 ^a^	31.13 ± 4.55 ^b^	37.76 ± 1.26 ^b^	35.98 ± 3.74 ^b^	28.29 ± 1.56 ^a^

Data were expressed as mean ± SD. Different superscripts indicate significance at *p* < 0.05. PC: pond culture. RC: rice–crayfish co-culture. LC: lotus–crayfish co-culture. CC: crab and crayfish co-culture. IC: industrial crayfish culture, SFA: saturated fatty acid, MUFA: monounsaturated fatty acid and PUFA: polyunsaturated fatty acid.

## Data Availability

The data that support the findings of this study are available from the corresponding author upon reasonable request.

## Supplementary Materials

The following supporting information can be downloaded at: https://www.mdpi.com/article/10.3390/biology15110875/s1. This file includes Figure S1: A view of the industrial culture site for *Procambarus clarkii*; Table S1: Composition of the feed for industrial culture of *Procambarus clarkii* (dry matter basis, g/kg). Table S2. PERMANOVA results of metabolic profiles among different culture modes.
