# Supplementary material for: The Effects of Different Culture Modes on the Nutritional Quality of Procambarus clarkii and Mechanistic Insights: A Metabolomic Perspective
Source: Biology (Basel). 2026 Jun 2;15(11):875. doi: 10.3390/biology15110875 (PMC13255845; doi:10.3390/biology15110875)
Supplement: Supplementary file 1 [file biology-15-00875-s001.zip › biology-4301924-supplementary.pdf]

Supplementary data for

The effects of different culture modes on the nutritional quality of *Procambarus clarkii* and mechanistic insights: a metabolomic perspective

Ting Liu[a,b], Juan Tian[a], Lang Zhang[a], Jianwu Chen[a], Yali Yu[a], ChenTian[a] and Jinhua Gan[a]*

[a]*Yangtze River Fisheries Research Institute, Chinese Academy of Fishery Sciences, Wuhan, 430223, China*

[b]*Freshwater Fisheries Research Center, Chinese Academy of Fishery Sciences, Wuxi, 214081, China*

* Corresponding author at: Yangtze River Fisheries Research Institute, Chinese Academy of Fishery Sciences, Wuhan, 430223, China

E-mail address: gjh@yfi.ac.cn (Jinhua Gan)

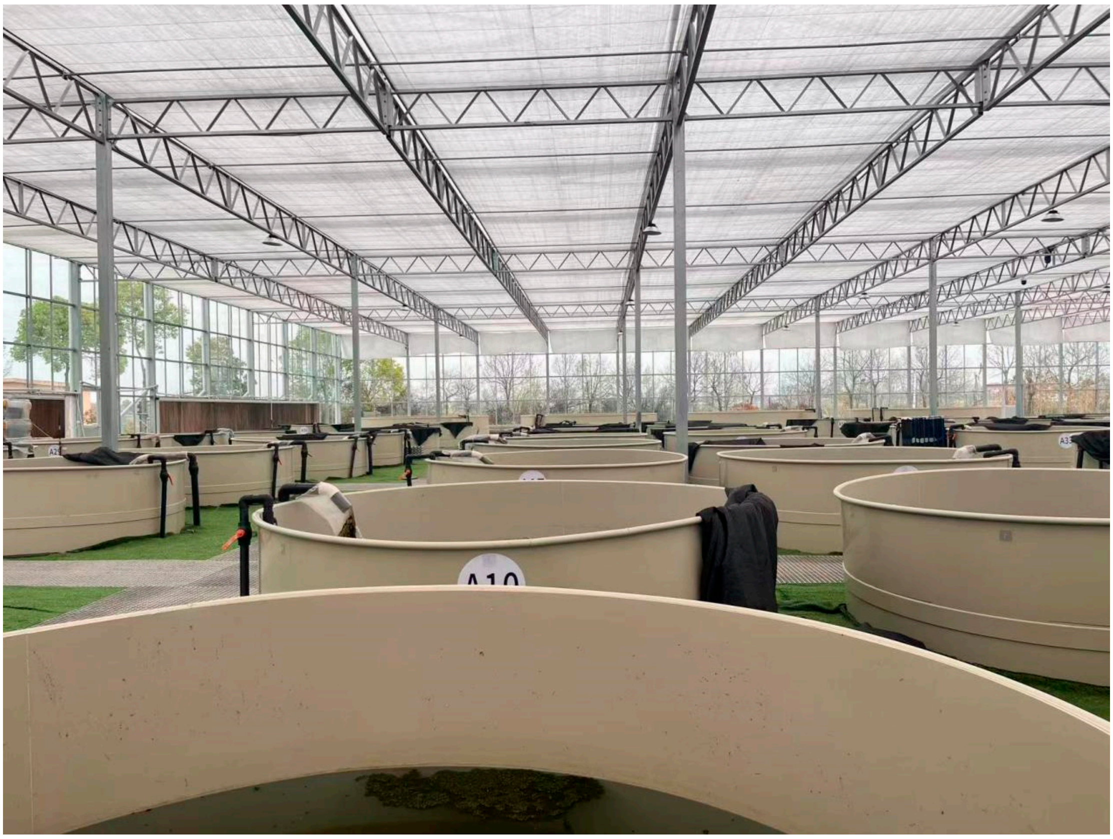

Figure S1. A view of the industrial culture site for *Procambarus clarkii.*

Table S1. Composition of the feed for industrial culture of *Procambarus clarkii* (dry matter basis, g/kg)

Ingredients	Dry matter basis (g/kg)
Fish meal	80.00
Gelatin	50.00
Wheat flour	320.00
Sodium alginate	20.00
$Ca(H_2PO_4)_2$	30.00
Yeast extract	20.00
Fish oil	27.50
Soybean oil	27.50
Soybean lecithin	10.00
Vitamin premix[a]	10.00
Cholesterol	5.00
Mineral premix[b]	10.00
Vitamin C	3.00
Vholine chloride	2.50
Chitosan	2.00
Astaxanthin	0.50
Bentonite	50.00
Micro-cellulose	149.50
Crystalline amino acid premix	182.50

[a]：vitamin premix contains vitamin A 4 g/kg, vitamin D 0.02 g/kg, vitamin E 10 g/kg, vitamin K3 10 g/kg, vitamin B1 10 g/kg, vitamin B2 10 g/kg, vitamin B6 20 g/kg, nicotinic acid 40 g/kg, biotin 0.2 g/kg, calcium pantothenate 20 g/kg, folic acid 0.5 g/kg, vitamin B12 0.01 g/kg, vitamin C 20 g/kg, inositol 400 g/kg, all ingredients were diluted with micro-cellulose to 1 kg.

[b]：mineral premix contains KIO_3 0.6 g/kg, $Na_2SeO_3 \cdot 5H_2O$ 0.08 g/kg, KH_2PO_4 320 g/kg, $MgSO_4$ 200 g/kg, $MnSO_4 \cdot H_2O$ 20 g/kg, $CuCl_2 \cdot 2H_2O$ 2 g/kg, $ZnSO_4 \cdot 7H_2O$ 60 g/kg, $FeSO_4 \cdot 7H_2O$ 50 g/kg, NaCl 100 g/kg, $CoCl_2 \cdot 6H_2O$ 2 g/kg, all ingredients were diluted with micro-cellulose to 1 kg.

Table S2. PERMANOVA results of metabolic profiles among different culture modes

Source	DF	SS	MS	Pseudo-F	P-value
Group	4	18.726	4.6815	7.842	0.001
Residual	25	14.930	0.5972	/	/
Total	29	33.656	/	/	/

Notes: PERMANOVA was performed based on Bray-Curtis distance with 999 permutations. DF, degrees of freedom; SS, sum of squares; MS, mean square.
